# Learning a Weighted Meta-Sample Based Parameter Free Sparse Representation Classification for Microarray Data

**DOI:** 10.1371/journal.pone.0104314

**Published:** 2014-08-12

**Authors:** Bo Liao, Yan Jiang, Guanqun Yuan, Wen Zhu, Lijun Cai, Zhi Cao

**Affiliations:** Key Laboratory for Embedded and Network Computing of Hunan Province, the College of Information Science and Engineering, Hunan University, Changsha Hunan, China; National Institute of Genomic Medicine, Mexico

## Abstract

Sparse representation classification (SRC) is one of the most promising classification methods for supervised learning. This method can effectively exploit discriminating information by introducing a 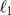 regularization terms to the data. With the desirable property of sparisty, SRC is robust to both noise and outliers. In this study, we propose a weighted meta-sample based non-parametric sparse representation classification method for the accurate identification of tumor subtype. The proposed method includes three steps. First, we extract the weighted meta-samples for each sub class from raw data, and the rationality of the weighting strategy is proven mathematically. Second, sparse representation coefficients can be obtained by 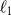 regularization of underdetermined linear equations. Thus, data dependent sparsity can be adaptively tuned. A simple characteristic function is eventually utilized to achieve classification. Asymptotic time complexity analysis is applied to our method. Compared with some state-of-the-art classifiers, the proposed method has lower time complexity and more flexibility. Experiments on eight samples of publicly available gene expression profile data show the effectiveness of the proposed method.

## Introduction

The development of high-throughput technologies has enabled scientists to monitor the gene expression levels in tens of thousands of genes simultaneously in a single experiment. This technology has become a symbol of the post-genomic era [1]. Biomedical research indicates that tumor development is related to the change in gene expression levels and that tumor-related biomarkers are usually associated with a few genes. Thus, identifying tumor tissue or disease-related biomarkers accurately is of great practical significance. However, gene expression profile data are characterized by very high dimensionalities and small sample size. The curse of dimensionality problem makes classification challenging.

Some dimensionality reduction methods have recently been proposed to solve the “large 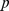, small 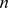
” problem [2]. Feature extraction and feature selection are two methods of dimensionality reduction; feature extraction transforms original features (genes) into a set of new features by subspace learning [3]–[5]. However, suitable biological interpretation is difficult to obtain from the subspace learning dimensionality reduction results. Feature selection is another commonly used dimensionality reduction method that selects a sub-set of genes that can best predict the response values from the raw data [6]. Although dimensionality reduction can significantly improve computational efficiency, this process can easily lead to over-fitting when a classifier is applied.

Sparse representation classification (SRC) was proposed by Wright et al. [7] for face recognition. With 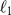 sparsity constraint, a testing face can be approximately represented by parts of the training data that are from the same class. Unlike traditional classification methods such as support vector machine and 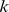 nearest neighbor classifier, SRC is robust to both noise and outliers. However, the orginal training samples may not contain suffiient discriminating information compared with meta-samples [8].

To capture more alternative information from gene expression data, the so-called meta-samples are proposed by [8]–[11]. These samples can be regarded as a set of bases, the linear representation of which can represent the training data. In [11], penalized matrix decomposition is used to extract meta-samples, and clustering is performed on those meta-samples. In [8], the meta-sample based sparse representation classification (MSRC) method is proposed. This method is robust to over-fitting problem and noise. However, MSRC needs two predefined parameters, namely, the number of meta-samples and the sparse penalty factor. These two parameters are data dependent. Thus, model selection methods, such as cross-validation (CV), significantly affect the classification results. In this study, we propose a non-parametric version of MSRC to address this optimal parameter selection problem. The main contributions of this paper are as follows:

1. The data-dependent sparsity can be automatically adjusted, rather than empirically chosen. Without computationally expensive model selection, our method is scalable and efficient.
2. The existing MSRC [8] method requires the appropriate selection of the number of meta-samples for each sub class, which is a laborious task. We address this problem by introducing a simple weighting strategy for the meta-sample of each category, and the rationality of weighting strategies is mathematically proved.
3. Extensive experiments are performed to evaluate the proposed method. Experimental results show the superiority of the non-parametric version of MSRC compared with some state-of-the-art classifiers. Section 3 presents more details.

The remainder of this paper is organized as follows: prior work on sparse representation classification and the fundamentals of the proposed method are described in Section 2. Section 3 presents the experimental results. The proposed method is discussed in Section 4. Section 5 concludes this paper.

## Methods

This study primarily aims to establish the manner by which to devise an robust classifier for tumor subtype classification. Given a microarray data set 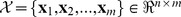 and a set of class labels 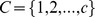, 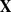 is a matrix with 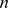 rows and 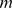 columns. Each column of 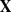 denotes a sample, whereas each row of 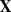 denotes a gene. Let 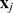 denote the 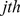 sample, which is a column vector with 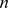 dimensional. For each element in 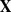, 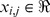 denotes the expression level of the 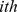 gene in the 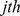 sample. We provide a summary of the abbreviations used in this study in Table 1. For clarity, we use boldface and lowercase type letters for vectors and boldface and capital type letters for matrices.

**Table 1:** Notations and abbreviations used in this paper.

Notation	Description
SVD	Singular value decomposition
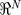	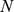 dimensional real number vector
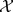	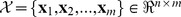 denotes gene expression data set with 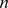 genes, 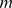 samples
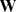	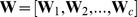 meta-samples associate with 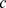 classes
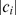	Number of samples belong to class 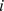
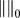	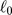 norm
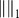	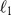 norm
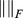	Matrix Frobenius norm

Gene expression profile data are high-throughput data with tens of thousands of genes. However, the number of samples is usually very small, which makes classification challenging. To avoid the curse of dimensionality, differential gene expression analysis [12], [13] is widely used to exclude redundant and irrelevant genes before classification. In our study, we use the Relieff [14] method to select a subset of informative genes for further analysis. In the following subsections, we briefly review meta-sample and sparse representation classification. we then propose weighted meta-sample based parameter free sparse representation classification (PFMSCR).

### Meta-samples versus gene expression samples

**Figure 1:**
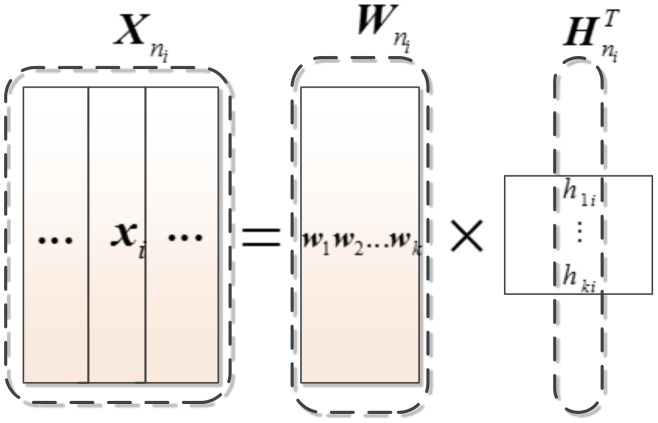
Illustration of meta-sample model: each column vector of 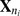 can be represented within a linear combination of meta-samples in 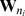, and the column of 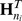 corresponds to the linear combination coefficients.

As illustrated in Figure 1, meta-samples can be regarded as basis samples that contain the essential information of the original data. A given testing sample can be represented by a linear combination of meta-samples from the same class. Concretely, suppose 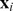 is associated with the 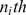 class, where 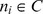, and the 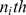 class samples in the training data have 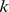 meta-samples, namely, 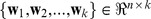. Sample 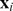 can be formulated as Eq. (1). 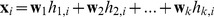
(1)


Mathematically, meta-samples extraction can be regarded as a type of matrix decomposition, including non-negative matrix factorization [15], singular value decomposition (SVD) [16], and principal component analysis [17], where matrix 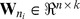, and 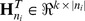 denote the meta-sample and meta-gene, respectively. In singular value decomposition, 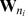 is a maximum linearly independent group of 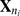 column vectors.

Biologically, meta-samples are also called eigenarray [18] or basis snapshot for gene expression data. Han et al. [17] used meta-samples to identify tumors from microarray data and found that meta-sample-based classification can effectively avoid over-fitting. Zheng et al. [10], [11], [18] proposed a novel cluster method based on meta-samples, which meta-samples can be regarded as cluster indictors.

Prior works revealed that meta-samples preserve some desired discriminant information of samples from the same class.

### Sparse representation classification problem revisited

In this subsection, we revisit the sparse representation problem briefly. Sparse representation is one of the most important components of machine learning and data mining community that has wide applications in such fields as text mining, image classification, and bioinformatics. In this work, we interpret the sparse representation problem from the view of linear algebra.

From the standpoint of linear equations system 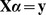, the solution of 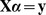 has three possible states:

1. Linear equation systems have infinitely many solutions if they are underdetermined (i.e., 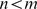).
2. Linear equation systems have a unique solution if they are well posed.
3. Linear equation systems have no solution if overdetermined (i.e., 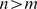).

In the first scenario, one can pursue the sparse solution by regularization [19]. The problem can be formulated as 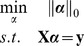
(2)


However, 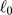 norm is an NP-hard combinational optimization problem, and difficult to solve, fortunately, 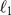 norm is an appropriate convex approximate to 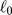 [20]. If the solution is sparse enough, 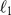 minimization is equivalent to 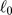 minimization [21], such that we can reformulate Eq. (2) as
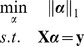
(3)


For the other two scenarios, the sparsity of 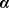 cannot be guaranteed. However, one can still obtain a sparse solution by adding a penalty term that shares the same formulation as LASSO [22]

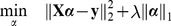
(4)


Compared with Eq. (3), Eq. (4) is an unconstrained convex problem. Notably, 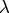 makes a tradeoff between sparsity and regression error and should be empirically chosen. A larger 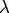 yields a sparser 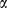. However, one might run the risk of increasing regression error term 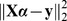.

Sparse representation assumes that a signal can be reconstructed by a small number of basis signals within a linear combination. Thus, Eq (3) can be named as basis pursuit [23]. In bioinformatics applications, one can suppose that a testing sample can be well reconstructed by the training data from the same class within a linear combination, which is a very useful assumption for our later work.

### Meta-sample based sparse representation

Zheng et al. [8] proposed MSRC method to predict tumor subtypes. In such situations, 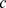 classes of meta-samples are extracted, denoting as 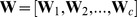 with the same classes being conjoined together, where meta-samples are column vectors (two kinds of meta-sample are proposed in [8]). Given a test sample 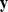 associated with class 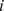, MSRC tries to find sparse reconstruct coefficients in terms of all meta-samples using Eq. (4). In particular, [8] tries to solve the sparse representation problem using 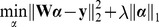. In ideal cases, the nonzero entries in 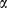 will only be associated with the 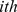 class meta-samples of 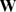, as shown in Eq. (5). 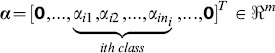
(5)


**Figure 2:**
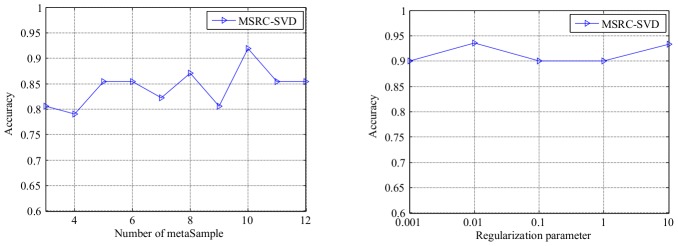
Optimal classification accuracy of MSRC achieved on COLON; the 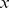-axis represents the number of meta-samples (left) and the regularization parameter (right). Classification accuracy is more sensitive to the number of meta-samples rather than to the regularization parameter.

Notably, the gene expression profile contains data with high dimensionality and small sample size (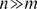). The sparsity can only be achieved by adding a penalty term. However, the optimal number of meta-samples and penalty factor 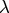 are essentially important in classification applications. Figure 2 illustrates that if the meta-samples are improperly set, the prediction accuracy of MSRC drops seriously on COLON dataset. Specifically, in the left part of Figure 2 shows that the 10-fold stratified cross validation classification accuracy is achieved by varying the number of meta-samples from 3 to 12 for each subclass. We can observe that the performance is less sensitive to various regularization parameters within the scope of 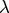 from the right part of Figure 2. Thus, model selection is essential and laborious work on different data sets.

To overcome this weakness, this study proposed a novel parameter free meta-sample based sparse representation classification (PFMSRC) method.

### Parameter free meta-sample sparse representation (PFMSRC)

In this subsection, we first propose a heuristic weighted strategy, the reasonableness of which is theoretically proven. We then construct an underdetermined linear equation system, in which the data-dependent sparsity can be self-adaptively tuned by 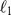 norm regularizer.

Let 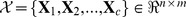 be gene expression profile data, with the same classes being conjoined together, that is, 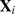 contains all samples associated with the 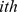 class. We factorize 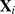 by performing SVD. The singular values are sorted in descending order 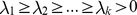, where 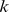 is the column rank of 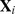, and 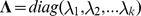 denotes diagonal matrix with singular values being diagonal elements. One can extract weighted meta-samples associated with class 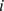 as 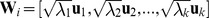, where 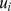 is a column vector in 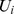, and 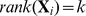.
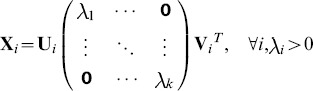
(6)


Alternatively, Eq. (6) can be compactly reformulated as 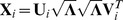. This weighting scheme can enhance the influence of main singular vector in 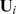. That is, larger 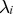 makes the associated meta-sample more important. Moreover, the weighting scheme works well in the following experiments. Compared with [8], Zheng et al. extracted meta-samples by performing SVD as well. However, in their algorithm framework, the number of meta-samples used for classification is determined during the cross-validation step. On the contrary, PFMSRC tries to avoid the cross-validation part by weighting the all meta-samples and weakening the influence of minor eigenvectors rather than using several of them for classification. Proposition 1 theoretically proves the reasonableness of the weighting strategy in measuring the importance of each metasample.

**Proposition 1.** *Singular value is a reasonable weighting factor for measuring the importance of meta-samples.*

*Proof.* Let 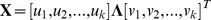, where 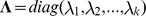 and 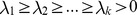, 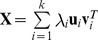, considering evaluation metric function 
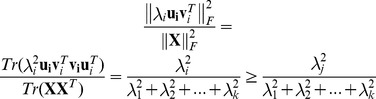
, one can conclude that 
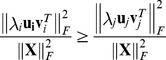



This completes the proof. □

The evaluation metric function is used to measure the meta-sample's contribution of the meta-sample to the raw data reconstruction in terms of 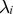. 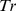 denotes matrix trace. Note that, functions 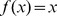 and 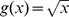 have the same monotonicity, which makes the weighting strategy reasonable.


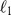
 graph was proposed by Cheng et al. [24] to measure the similarity among samples. Inspired by their work, sparsity can be obtained by 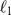 regularizer on underdetermined linear equation systems. Concretely, a testing sample can be recovered by weighted meta-samples within a linear combination with a noise term added, formulated as Eq. (7)
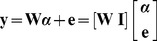
(7)


Let 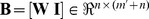 and 
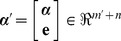
, where 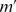 represents the number of meta-samples corresponding to 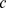 classes, 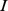 is an identity matrix, and 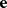 is the noise term. Alternatively, one can solve the following minimization problem:
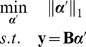
(8)


Theorem 1 proves that Eq. (8) is a underdetermined linear system. As stated in Subsection 2.2 the sparsity of underdetermined linear system can be automatically tuned by 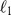 regularization (the first scenario). Moreover, (8) is a canonical convex problem with equality constraints, which can optimize sparse representation coefficients and noise term simultaneously. The globally optimal solution can be efficiently solved by CVX package [25] in polynomial time. Notably, the package solves the optimization problem by dualization rather than interior point method because the former is significantly faster than the latter.

**Theorem 1.** *Linear equation system (8) is underdetermined, and* 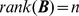.

*Proof.* We can find a sub matrix in 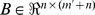, such as 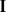 and 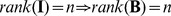. This completes the proof. □

**Figure 3:**
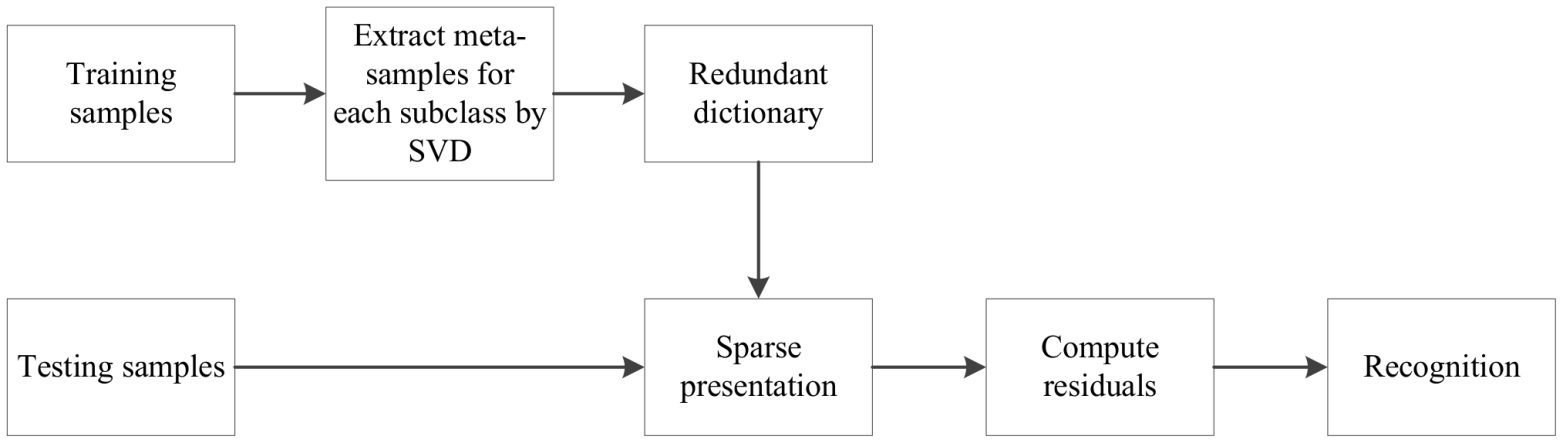
The flowchart of PFMSRC scheme.

Note that 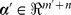 is a sparse vector with 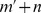 entries. The first 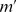 components correspond to linear representation coefficients, whereas the last 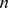 components characterize model noise or regression error. However, the test sample 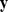 from one of the classes in training data cannot be well reconstructed by meta-samples associated with the same class in most instances because of the existence of noises. Figure 3 illustrates the flowchart of our PFMSCR scheme, the redundant dictionary is constructed by combining meta-samples and noise term.

We define a projection function 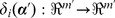 for each class 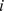, which selects the coefficients associated with the 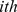 class from the first 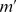 components in 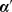, whereas the other entries are appropriately padded with zeros in 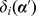. The reconstruction relationship 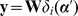 is not always holden. However, the minimized reconstruction error criterion 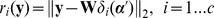 is a good approximation to classify testing samples. We summarize the proposed classification method as follows.

Step 1. Input training sets 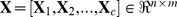, class number 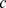, and testing sample 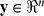;

Step 2. Normalize training set samples and testing sample to obtain unit 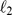-norm;

Step 3. Extract weighted meta-samples 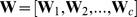 for each class (meta-samples with the same class are conjoint);

Step 4. Solve non-parametric sparse representation problem by Eq. (8);

Step 5. Compute residuals for each class 
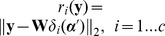
;

Step 6. Return class label of 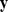 as 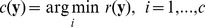;

PFMSRC can be considered as a non-parametric version of MSRC, compared with the former having the following merits:

1. The weighted meta-samples are orthogonal with one another. That is, no redundancy exists among meta-samples, and the weight enhances the influence of the main singular vector, such that discriminant information can be well retained.
2. The data-dependent sparsity can be automatically tuned without human intervention. Thus, PFMSRC has better scalability and robustness.
3. The time complexity of PFMSRC is lower than that of MSRC, since computationally expensive model selection work need not be accomplished for parameter optimization. Time complexity can be estimated as: weighted meta-sample extraction step needs time complexity 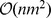, 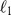 minimization needs time complexity 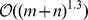, the total complexity for PFMSRC is 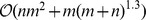.

In the following section, we will conduct extensive experiments on micoarray data to evaluate the effectiveness of our scheme, and microarray data repository information as well as the accession number is given by Table 2.

**Table 2:** Descriptions of microarray data repository and the accession number.

Datasets	Repository	Accession number
Colon	Gene Expression Omnibus	GDS4379
Acute leukemia data	Gene Expression Omnibus	GSE19475
DLBCL	Gene Expression Omnibus	GSE15177
Gliomas	Gene Expression Omnibus	GSE54792
SRBCT	Gene Expression Omnibus	GSE1825,GSE31186,GSE31217
ALL	Gene Expression Omnibus	GSE23024
MLLLeukemia	Gene Expression Omnibus	GSE11038
LukemiaGloub	Gene Expression Omnibus	GSE10283

## Experiments

In this section, we will evaluate the performance of the proposed PFMSRC algorithm against four state-of-the-art algorithms, namely, linear discriminant analysis (LDA+SVM), independent component analysis (ICA+SVM), SRC, and meta-sample sparse representation (SVD-MSRC). The former two are model based and accompanied by feature extraction. These two algorithms are regarded as baseline. For the model-based method, support vector machine [26], [27] with radial basis function kernel is employed as a classifier. The experiments are performed on four binary-class classification data sets and four multiclass classification data sets. All experiments are implemented in Matlab environment and run on a personal computer with intel Pentium4 dual core CPU 2.4 GHZ and 4 G RAM. The summarized descriptions of the eight gene expression profile datasets are provided by Table 3.

**Table 3:** Data set descriptions.

Datasets	Samples	Genes	Subclass number
Colon	62	2000	2
Acute leukemia data	72	5000	2
DLBC	77	7129	2
Gliomas	50	12625	2
SRBCT	83	2308	4
ALL	248	12626	6
MLLLeukemia	72	12582	3
LukemiaGloub	72	7129	3

- Colon [28] consists of 62 samples with two subclasses including 40 tumor and 22 normal samples. The highest 2000 genes with minimal intensity in the tissues are retained from the original of more than 6500 genes. This dataset can be downloaded from [29].
- Acute leukemia data [30], consist of 72 samples with two subclasses, including 47 acute lymphoblastic leukemia patients and 25 acute myelogenous leukemia patients. Each sample contains 7129 genes. This dataset can be downloaded from [29].
- DLBCL [1] consists of 77 samples with two subclasses, including 58 diffuse large b-cell lymphoma samples and 19 follicular lymphoma samples. Each sample contains 7129 genes. This dataset can be downloaded from [31].
- Gliomas [32] consist of 50 samples with two subclasses (Glioblastomas and Anaplastic Oligodendrogliomas), and each sample contains 2308 genes. This dataset are available at [31].
- SRBCT [33] consist of 83 samples with four subclasses (Ewings sarcoma, Burkitts, Neuroblastoma and rhabdomyosarcoma). Each sample contains 2308 genes. The datasets are available at [31]
- ALL [34] consists of 248 samples with six subclasses. Each sample contains 12626 genes. The datasets are available at [31].
- MLLLeukemia [35] consists of 72 samples with three subclasses. Each sample contains 12582 genes. The datasets are available at [29].
- LukemiaGloub [30] consists of 72 samples with three subclasses. Each sample contains 7129 genes. The datasets are available at [31].

### Dataset preprocessing and experiment setup

Gene expression profiling involves data with high dimensionality and small sample size. The exclusion of redundant and irrelevant data is critical for classification. As suggested by [36], restaining only the top 400 genes makes a good tradeoff between computational complexity and biological significance. In our experiment, the top 400 genes are selected from each dataset by applying the Relieff [14] algorithm to the training set.

For LDA+SVM algorithm, we simply extract 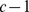 new features to train the classifier, as LDA can find at most 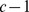 meaningful projection vectors in the subspace, where 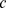 denotes the number of classes. SVM kernel parameters are determined by 10-fold cross-validation. In fact, the determination of the number of independent components is also an empirically dependent work. Here, we use the same method as suggested by [18].

SRC and MSRC methods need parameter 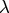 to control sparsity. MSRC also needs the number of meta-samples of each class as a key parameter. Each dataset 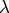 is searched from 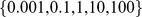 by 10-fold CV on training data, and the number of meta-samples for each class is set as recommended by [8].

### Experiments on binary classification problem

To evaluate the performance of five methods on a balanced split data set, we randomly select 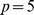 to 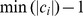 samples per subclass as training set and use the rest for testing to guarantee that at least one sample in each category can be used for test, 20 times training/testing are randomly split, and the average classification accuracies are presented. The best prediction accuracy is in boldface for each gene expression profile dataset.

**Figure 4:**
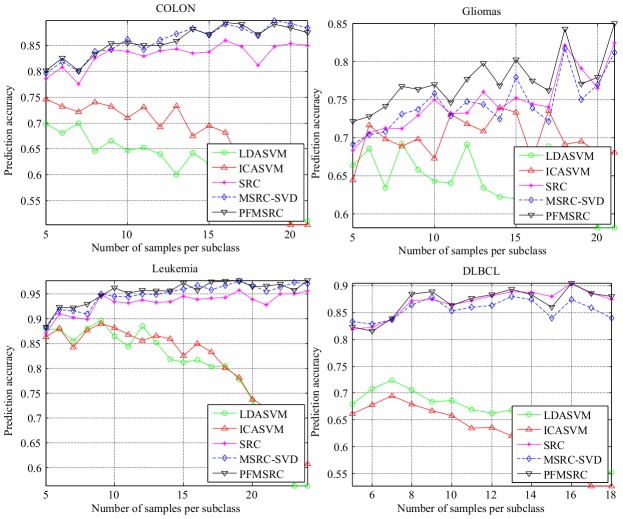
Comparison of prediction accuracy on four binary classification datasets by varying the number of samples from per subclass; when 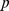 is larger than 10 the model based method prediction accuracy decreases as 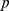 increases.

We show the average performance comparison on four binary classification tasks in Figure 4. PFMSRC exhibited encouraging performance. Although Gliomas was difficult for classification, the proposed approach can still achieve 85% classification accuracy via 20 samples per subclass used for training. Notably, the classification accuracy of LDA+SVM and ICA+SVM dropped quickly as more samples are taken for training; the same observations can be found in [36]. This fluctuation phenomenon can be interpreted as follows: (1) For the binary classification case, the feature extracted by LDA has only one dimension that is insufficient to capture the intrinsic discriminating information. Thus, model-based classification methods have difficulty in preventing the over-fitting phenomenon. (2) When evaluating the performance on the testing set the number of samples changes as more samples are used for training.

Classification accuracy, specificity, and sensitivity are some popular evaluation metrics. In this work, we use all three to evaluate performance, and the results are reported in Table 4, 5, and 6, respectively. The three methods can achieve satisfactory performance not only on the specificity metric but also on the sensitivity metric. Compared with SRC and MSCR, PFMSRC outperforms its competitors in most cases. A comprehensive consideration is that PFMSRC achieves the best performance, followed by MSRC and SRC.

**Table 4:** Comparison on four binary classification tumor data sets; for each data set, 10 samples per class are randomly selected for training and the rest are used for testing.

Dataset name	LDA+SVM	ICA+SVM	SRC	MSRC-SVD	PFMSRC
colon	74( 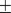 7.85)	64.55( 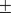 7.39)	84.20( 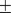 3.65)	84.20( 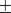 4.81)	**85.45(** 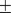 **3.33)**
DLBC	66.76( 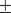 6.67)	68.33( 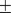 4.78)	**86.49(** 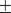 **3.39)**	85.35( 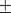 4.91)	86.40( 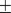 5.69)
Gliomas	65.83( 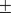 8.08)	69.83( 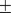 9.52)	75.00( 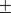 6.35)	75.83( 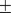 7.24)	**77.00(** 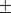 **6.48)**
Acute leukemia	89.71( 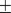 3.14)	89.13( 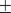 4.96)	93.46( 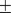 3.82)	94.52( 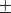 3.65)	**96.25(** 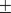 **2.20)**

We report the standard deviations in parentheses.

**Table 5:** Comparison of specificity by different methods on four binary classification data sets.

Dataset name	SRC	MSRC-SVD	PFMSRC
colon	90.00	**92.50**	**92.50**
DLBC	**96.55**	94.83	**96.55**
Gliomas	72.73	**77.27**	**77.27**
Acute leukemia	100	100	100

**Table 6:** Comparison of sensitivity by different methods on four binary classification data sets.

Dataset name	SRC	MSRC-SVD	PFMSRC
colon	81.82	**86.36**	**86.36**
DLBC	**1**	**1**	94.74
Gliomas	82.14	78.57	**89.29**
Acute leukemia	88.00	**92.00**	84.00

### Experiments on multiclass classification problem

**Figure 5:**
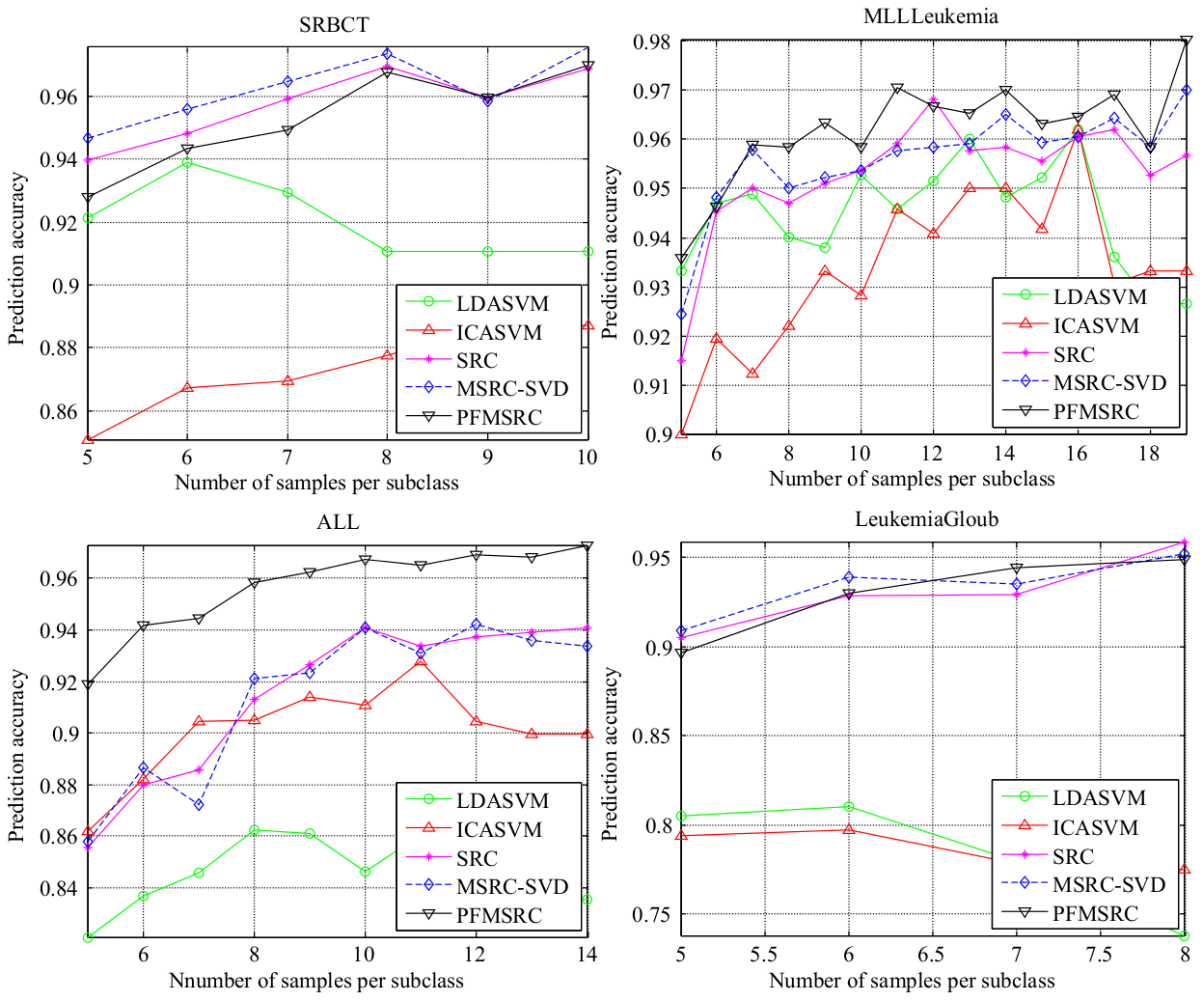
Comparison of prediction accuracy on four multiclass classification datasets by varying the number of samples from per subclass; when 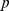 is larger than 10 the performance degradation of model based methods is less significant than that of binary classification.

We investigate multiclass classification performance on four publicly available data sets. The experimental setup is the same as that for the binary classification case. On one hand from Figure 5 and Table 7 it can be seen that (1) the classification accuracies of SRC, MSRC, and PFMSRC are increased on all multiclass classification datasets as more samples per subclass are taken for training. (2) ALL has six subclasses, and the proposed PFMSRC achieves the highest classification accuracy, which indicates that we have potential superiority on multiclass classification task. (3) LDA can capture more discriminating information on the multiclass classification task, and the over-fitting phenomenon is reduced compared with the binary classification task.

**Table 7:** Comparison on four multiclass tumor data sets; for each data set, 10 (8 for LeukemiaGloub) samples per class are randomly selected for training the rest are used for testing.

Dataset name	LDA+SVM	ICA+SVM	SRC	MSRC-SVD	PFMSRC
SRBCT	91.05( 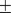 4.61)	88.72( 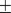 5.56)	96.86( 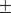 2.64)	**97.56(** 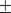 **3.06)**	96.98( 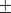 2.51)
ALL	86.12( 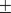 3.81)	91.38( 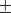 3.28)	94.07( 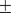 2.38)	94.07( 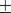 2.93)	**96.73(** 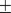 **1.68)**
MLLLeukemia	93.81( 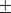 3.74)	93.33( 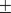 5.16)	95.36( 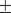 3.04)	95.36( 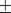 2.84)	**95.83(** 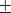 **2.88)**
LukemiaGloub	73.75( 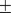 5.25)	77.50( 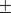 6.98)	**95.83(** 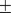 **2.14)**	95.21( 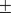 2.35)	94.90( 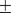 2.74)

The average accuracy and corresponding standard deviations are reported.

On the other hand, sparse representation based classification methods are less sensitive to the number of samples used for training model-based classification methods, which suggests a natural approach to select a classifier when the training sample size is small. Table 7 provides the performance description of the five classification methods. The proposed PFMSRC method performs consistently well with small standard deviations. On the SRBCT and ALL datasets, PFMSRC achieved 96.98% and 96.73%, respectively.

### Experiments with different number of genes

In this subsection, we evaluate the performance of the five methods with different feature dimensions on eight tumor data sets. For the training data, 10 samples per subclass are randomly selected, whereas the remaining samples are used for test. We perform the test with various numbers of genes, starting from 50 to 400 genes in steps of 20. The comparison experiment was performed 20 times, and the average prediction accuracy of our experiments on eight gene expression profile datasets was recorded for evaluation.

**Figure 6:**
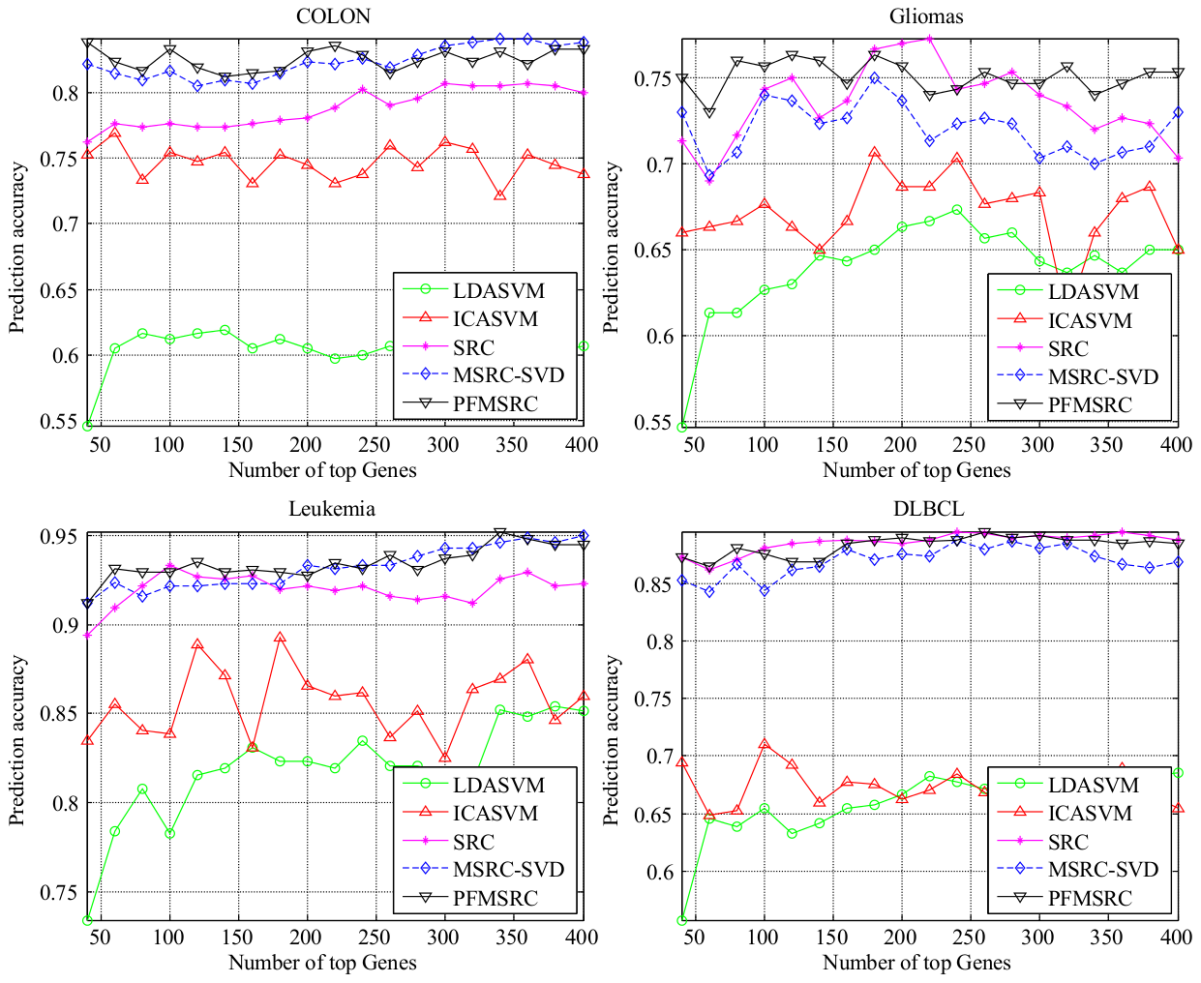
Comparison of prediction accuracy on four binary classification datasets by varying the number of top selected genes.

The balanced training sets for each dataset ensure fair evaluation as stated by [36]. The experimental result in Figure 6 shows that the proposed PFMRSC performs well when only 100 genes are used. We can observe the similar results in the multi-classification case as well.

In binary classification case, SRC, MSRC, and PFMSRC share the same curve trend. Compared with SRC and MSRC, PFMSRC performs well by using a smaller number of genes, SRC and MSRC can achieve comparable accuracy by using more genes. Evidently, SRC, MSRC, and PFMSRC consistently outperform LDA+SVM and ICA+SVM in all datasets.

**Figure 7:**
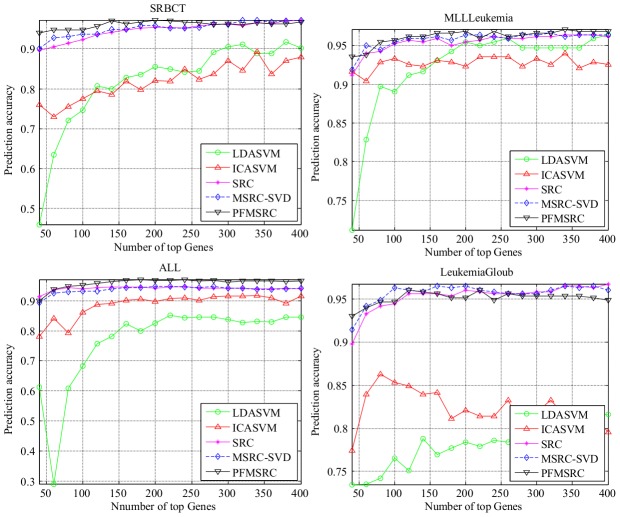
Comparison of prediction accuracy on four multiclass classification datasets by varying the number of top selected genes.

In the multiclass classification case, the performance of MSRC, SRC, and PFMSRC is very stable with respect to the number of genes, and all these methods converge fast to the optimal classification rate point. Figure 7 shows that compared with their performance in the binary classification case, SRC, MSRC, and PFMSRC are less influenced by gene dimension. Note that ALL is a multiclass dataset with six subclasses, but PFMSRC can still achieve a higher classification rate of 97% accuracy compared with SRC and MSRC. The same conclusion can be drawn for the SRBCT dataset.

In Table 8, we report the detailed classification accuracy. PFMSRC outperforms its competitors on most gene expression profile datasets, whereas SRC and MSRC-SVD perform the second best.

**Table 8:** The maximal average prediction accuracy of LDA+SVM, ICA+SVM, SRC, MSRC-SVD and PFMSRC on eight tumor microarray datasets.

Dataset name	LDA+SVM	ICA+SVM	SRC	MSRC-SVD	PFMSRC
colon	61.67	76.90	80.48	**84.05**	83.81
DLBC	68.07	71.05	**89.47**	88.42	89.47
Gliomas	67.33	70.67	75.33	75.00	**76.00**
Acute leukemia	85.38	88.85	93.27	95.00	**95.19**
SRBCT	91.16	89.30	**97.21**	**97.21**	**97.21**
ALL	85.16	91.44	96.46	93.59	**97.02**
MLLLeukemia	96.43	94.05	96.43	96.67	**97.14**
LukemiaGloub	81.63	91.81	94.79	94.68	**96.05**

### Comparsion of CV performance

To evaluate the classification performance on imbalanced split training/testing sets, we perform 10-fold stratified CV on tumor subtype dataset. All samples are randomly divided into 10 subsets based on stratified sampling: nine subsets are used for training, and the remaining samples are used for testing. This evaluation process is repeated 10 times, and the average result is presented. The 10-fold CV results are summarized in Table 9.

**Table 9:** 10-fold CV prediction accuracy of eight tumor microarray datasets using different classification methods.

Dataset name	LDA+SVM	ICA+SVM	SRC	MSRC-SVD	PFMSRC
colon	81.67	90.00	87.14	**90.24**	**90.24**
DLBCL	92.14	**97.14**	97.14	91.96	95.89
Gliomas	**86.50**	**86.50**	78.33	78.33	84.00
Acute leukemia	96.50	95.57	96.07	**97.50**	95.00
SRBCT	96.64	95.75	**1**	**1**	**1**
ALL	97.61	94.83	96.46	93.59	**97.63**
MLLLeukemia	95.65	95.89	**98.75**	**98.75**	97.32
LukemiaGloub	97.32	96.32	**98.57**	**98.57**	96.07

Table 9 shows that as the training sample size increases, the performance of these five classification methods is significantly improved. Model based methods LDA+SVM and ICA+SVM perform very well, with the classification accuracy increased significantly. In particular, the prediction accuracy of ICA+SVM ranges from 86.5% to 96.57% in all tumor expression profile datasets, which is comparable with those of SRC, MSRC and PFMSRC.

We can conclude that model-based approaches are more vulnerable to the small sample size problem, over-fitting should be resolved properly.

## Discussion

Based on the above experiments, we can draw the following observations:

1. Sparse representation based methods (SRC, MSRC, PFMSRC) consistently outperform the model-based methods (LDA+SVM, ICA+SVM) on all experiments. Especially, in balance splited datasets the prediction accuracy of model-based methods is significantly lower than that of sparse representation methods which may be attributed to the small sample size problem. However, SRC, MSRC, and PFMSRC perform well even when we take 5 samples per subclass for training and the rest for testing.
2. SRC, MSRC and PFMSRC are robust to various sample sizes and feature dimensions, as well as converge fast to the optimal classification rate. The experiments verify the results in [7], which favors the application of those methods. Note that, model-based methods (LDA+SVM, ICA+SVM) exhibit improved 10-fold CV classification accuracy. A reasonable explanation is that the over-fitting phenomena are dramatically reduced when 90% of original samples are used for training and the remaining 10% are used for evaluation in our experiments.
3. PFMSRC outperforms SRC and MSRC in most cases, which implies that the parameter free sparse representation and weighting strategies can capture more discriminating information, especially in multiclass classification. See Figure 5.
4. PFMSRC is a parameter-free method, in which the data dependent sparsity can be self-adaptively tuned, compared with SRC and MSRC in which search for a regularization parameter is laborious work. Moreover, the number of meta-samples is a key parameter for MSRC, as shown in Figure 2, which makes model selection more difficult.

## Conclusions

In this study, we proposed a novel non-parametric meta-sample-based sparse representation. The algorithm assumes that test samples can be well reconstructed within a linear combination of weighed meta-samples in the same class. We theoretically proved the rationality of the weighting strategy. A simple but efficient projection function is constructed by the sparse representation coefficients to complete the classification work. We also compare the performance of PFMSRC with that of two model-based methods and two sparse representation-based methods on eight tumor expression datasets. Experimental results have shown the superiority of the proposed method. We then drew some conclusions on the effects of both balanced split and imbalanced split testing/training sets on tumor classification problems.

PFMSRC exhibits stable performance with respect to different training sample sizes and feature dimensions compared with the other four algorithms. Thus, the extension of the sparse representation with dimensionality reduction (feature selection or feature extraction) in a unified framework is one of our future works.
